# Supplementary material for: The Impact of Sport Activity Shut down during the COVID-19 Pandemic on Children, Adolescents, and Young Adults: Was It Worthwhile?
Source: Int J Environ Res Public Health. 2022 Jun 28;19(13):7908. doi: 10.3390/ijerph19137908 (PMC9265880; doi:10.3390/ijerph19137908)
Supplement: Supplementary file 1 [file ijerph-19-07908-s001.zip › ijerph-1773602-supplementary.pdf]

Supplementary Methods

Materials and methods (extended version)

Study design and sampling

Between June and September 2021, a national retrospective cross-sectional survey of children, adolescents and young adults (aged ≤25 years) who used to play sports both at a competitive level (but not professional), as well as amateur, was performed. The survey was conducted using Google Forms web survey platform and the link was shared throughout social media and sport clubs and associations, including especially: UISP Lombardia, FIN Veneto, CRESCERE SANI Onlus, Kids United asd, Commissione Medico Scientifica Federale and Commissione Federale Antidoping e Tutela della Salute (FGIC), CSI, G.S. Muraglia Calcio Pesaro, Nuova Lodi and S.S. Turris Calcio. The first page of the questionnaire included a brief description of the study and the declaration of anonymity and confidentiality, along with the request to provide informed consent to proceed. The study was conducted in agreement with the national and international regulations and the Declaration of Helsinki (2000). The study did not require approval by the Ethics Committee because the online survey was anonymous and it was not possible to track sensitive personal data. The self-compiled questionnaires were transmitted to the Google platform and the final database was downloaded as a Microsoft Excel sheet.

Questionnaire

The questionnaire was developed by a panel of experts with in-depth knowledge on different areas, among which epidemiology, nutrition and psychology. It was in Italian and required an estimated time of 12-15 minutes to be completed. It comprised 77 mainly non-mandatory items. The two first questions investigated age and sport practiced: responders older than 25 years and professional athletes were not allowed to proceed with the questionnaire basing on our exclusion criteria. The questionnaire included multiple choice and open-ended questions divided into six different sections:

sport-related questions, SARS-CoV-2 infection, socio-demographic, physical activity and mental health, diet, screen time. General survey administration was anticipated by a two-weeks pilot phase in which we tested the reliability of the adopted questionnaire and clarity of the questions. The original version of the questionnaire is provided (Supplementary File 1).

Participants were first asked whether they had practiced a sport activity in the period between September 2020 and May 2021. If yes, they answered to several sport-related questions, including: sport type, number and two-weeks period of training sessions, participation to sport competitions, participation to sport activities organized by Sport Society or Sport Centers, individual/team training, indoor/outdoor activities, preventive measures (multiple choice among none, distancing, face masks during training, environment disinfection, aeration, use of lockers and showers, triage, other to be specified). The section of SARS-CoV-2 infection included questions on the number and date of positive test(s) for athletes and for their cohabitants and total number of SARS-CoV-2 tests for athletes. Socio-demographic questions included gender, age, geographical area, height and weight in September 2020 and May 2021, school attendance, number of cohabitants, parents' education, parents' worksite, presence of outdoor spaces at home, outdoor physical activity. Physical activity and mental health section included an adaptation of the International Physical Activity Questionnaire (IPAQ) in which physical activity was referred to the period of Covid-19 picks instead of the last week. This questionnaire included different questions according to the age of responders, as required [1]. It consists of questions that record the frequency and duration of mild, moderate, and strenuous exercise performed during free time measuring physical activity and inactivity. It is a validated self-report measure of exercise that has been reliably used in previous studies [2]. Relevant psychological aspects were investigated using validated questionnaire, but these data were not included in the present analysis. Food consumption was investigated using a 16-items questionnaire [3] that measures the daily or weekly intake of the main food groups and adherence to the Mediterranean diet in the study period. Finally, we asked for the screen time, including television, personal computer, tablet, smartphone and videogames.

Patient and public involvement

General survey administration was anticipated by a two-weeks pilot phase in which we tested the reliability of the adopted questionnaire and clarity of the questions by administration to the first version of the questionnaire to a small sample of 15 athletes and their families. We collected their feedback and minor adjustments were made to wording. Moreover, most of the adolescents and young adults suggested shortening the questionnaire to increase the compliance. We therefore eliminated some questions and decided to ask for willingness to answer to not mandatory sections (see questionnaire in Supplementary File 1 for details). We also use this pilot phase to estimate the time necessary to answer each section, in order to give more reliable indication in the online questionnaire. Preliminary research findings had been presented to a public congress organized by UISP Lombardia in November 2021, and participant sport societies and athletes were invited to participate. This presentation was disseminated through newspapers, social media and email. The final results included in the present paper will be disseminated to all participants by using similar strategy.

Statistical Analysis

To assess the association between sport activity and SARS-CoV-2 positivity during the study period we categorized sport activity into two ways. In the first ("training by closure period"), we grouped athletes based on whether they did sport activity during closure period (November 2020-March 2021), only in opening periods (September/October 2020 and April/May 2021) or did not do sports activity in the whole period. In the second ("weekly training sessions"), we considered the average number of weekly trainings (categorized as zero, up to two and more than two). Closure period definition is based on the observation that in Italy, non-professional sports were shut down or limited from start of November 2020 to the end of March 2021, depending on the local ordinances. Some sports activities were restarted in most Italian regions from April 2021, while others remained prohibited and/or limited. During "sport activities lockdown", in fact, it was allowed to continue practicing sport in specific circumstances, depending on the type of sport ("agonist" vs "amateur"), and the indication

from the different sport societies and local authorities. We graphically verified that “closure period” corresponded to an average decrease of sport participation in our database (Supplementary Figure 1). Chi Square test was performed to investigate the association between any SARS-CoV-2 positivity and study characteristics of the responders for categorical variables, while the non-parametric Wilcoxon-sum rank test was used for continues variables. At multivariable analysis, we applied unconditional logistic regression models to the data and calculated Odds Ratio (OR) with 95% Confidence Interval (CI) for the risk of being tested positive for SARS-CoV-2 at least once during the study period. Beyond sport participations and main demographic variables (age, expressed as school attendance, and gender), other covariates included in the model were the ones which resulted significantly associated with SARS-CoV-2 positivity at univariate analysis and remained statistically significant in multivariable analysis. A missing indicator variable was included for the categorical covariates with less than 20% of missing data, while covariates with more than 20% of missing data were not included in the multivariable model. In order to investigate the robustness of the results, we also performed a generalized linear models analysis for time point SARS-CoV-2 positivity with repeated measures, which included all the results of participation to sport activity and SARS-CoV-2 positivity in each of the two-weeks periods investigated in the questionnaire. In this analysis, for each subject with a positive SARS-CoV-2 test, the presence of at least one training session in the two-weeks period prior the positive test and during the two-weeks period of positivity was considered. If at least one training session was reported in these periods, we considered the exposure to sport activity as “present”, otherwise we defined it as “absent”. A logit link was used for the models and OR were calculated with 95%CI. All the main analyses were also reported for at least one SARS-CoV-2 positivity in the family and for the subgroup of athletes who declared they have had practiced sport activities organized by a Sport Society/Center.

Among the athletes who participated to any sport activity during the study period, we investigated the association between preventive measures and other sport-related characteristics with the risk of any SARS-CoV-2 positive test with unconditional logistic regression analysis.

BMI change was defined as the variation between the BMI calculated in September 2020 and the one in May 2021 using body weight and height reported by responders (measured as weight (kg)/height $(m)^2$)). The association between sport activity and responders' characteristics with logarithm of BMI change was assessed at univariate analysis with Wilcoxon sum rank test for categorical variables and Spearman correlation for continuous variables. At multivariable analysis we used a generalized linear model with logarithm of BMI change as dependent variable, including as covariate logarithm of baseline BMI and the variables associated to BMI change at univariate analysis (and then confirmed at multivariable analysis). The analysis was stratified for normal weight and overweight patients, defined according to age [4].

According to the WHO, children aged 5 to 17 years should exercise with an average of 60 daily minutes of moderate-to-vigorous PA. Adherence to the WHO guidelines as reported in[5] was defined based on athletes' age.

The Mediterranean adherence score was calculated according to the validation study [3]. It is assigned one point to participants reporting consumptions for each of the following foods that are characteristic of the Mediterranean diet: vegetables (≥2/day), fresh fruits (≥2/ day), dried fruits (≥2/week), whole grain cereals (≥1/day), pulses (≥2/week), fish (≥2/week) and olive oil intakes (≥3/day). We also assigned one point to those consuming red and processed meat ≤1-3/week. The total score range from 0 to 9.

All the tests were two-sided, with p-values<0.05 considered as statistically significant. The analyses were performed with R (version 4.1.1) and SAS (version 9.4) software.

Supplementary Figure S1. Number of subjects with at least one training session in different 15-days periods.

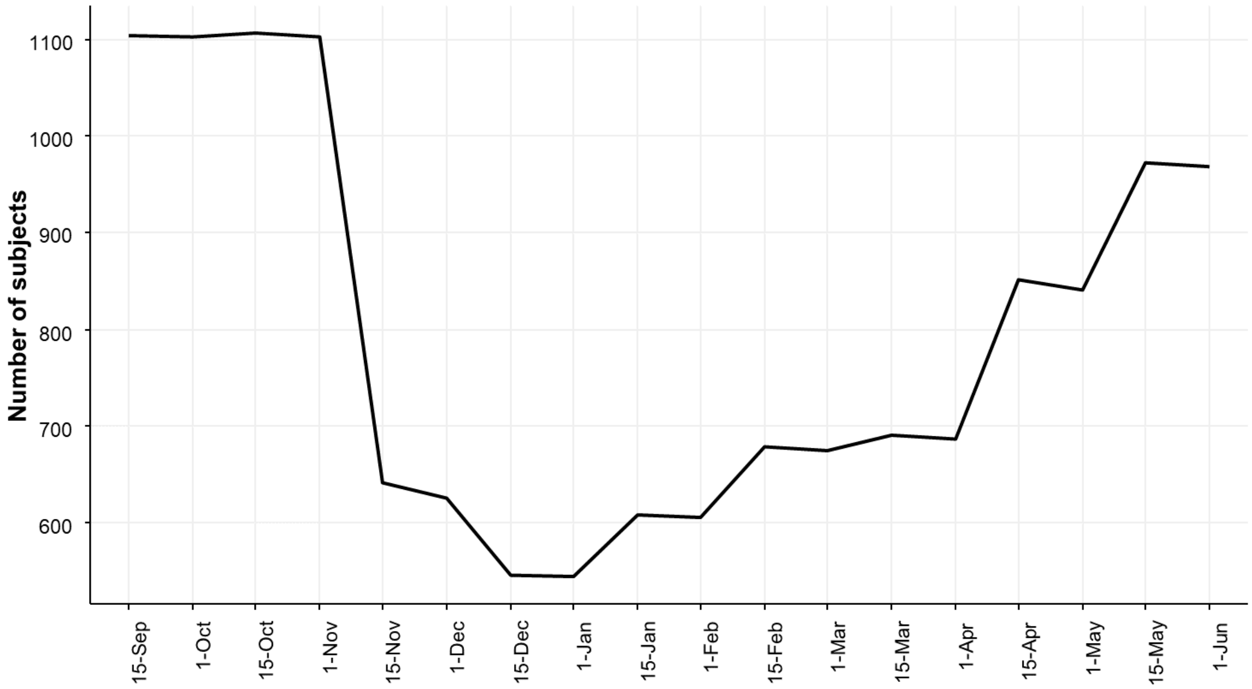

Supplementary Figure S2. Flow chart of the questionnaires included in the analysis.

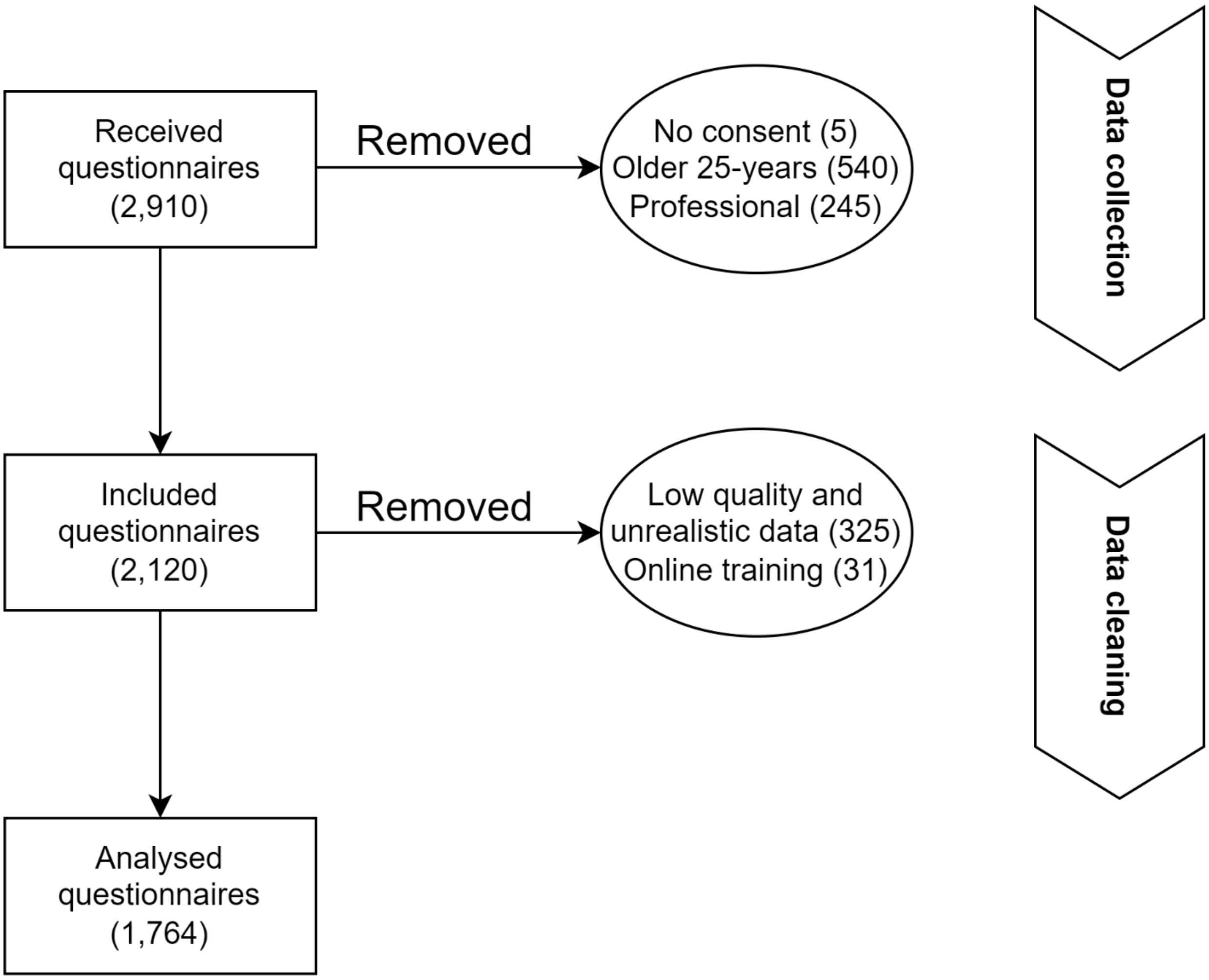

Note: low quality and unrealistic data refer to questionnaires with different age reported in different sections of the questionnaire, BMI out of realistic range (15-55), inexistent sports declared and bad words.

Supplementary Table S1. Additional investigated characteristics of the study population.

Characteristics	All (N=1,750)
**Adherence to WHO guidelines for physical activity**	
No	1143 (67%)
Yes	567 (34%)
**Number of cohabitants**[∞]	3 (2-4)
**Highest parents' education**	
Middle school or lower	50 (3%)
High school	560 (33%)
University or higher	1,112 (64%)
**Outdoor space at home**	
No	757 (43%)
Yes	978 (56%)
**Contact Sports**	
No	719 (45%)
Yes	960 (55%)
**Matches or competitions**	
No	1250 (71%)
Yes	499 (29%)
**Daily screen time**	
≤2 hours	437 (29%)
2-4 hours	404 (27%)
≥4 hous	659 (44%)

[∞]Median (Interquartile range)

Supplementary Table S2: Multivariable analysis for the risk of being tested positive for SARS-CoV-2 in the overall study population and in the subgroup of athletes enrolled in sports centers. Analysis comparing athletes with a different number of weekly training sessions.

	**Overall Analysis (n=1,732)**	**Enrolled in Sport Centers (n=1,669)**
	**OR [95% CI]**	**OR [95% CI]**
**Weekly training sessions**		
No	1.00 [reference]	1.00 [reference]
Up to two	0.88 [0.59, 1.30]	0.71 [0.48, 1.07]
More than two	0.99 [0.63, 1.55]	0.64 [0.39, 1.05]
**Gender**		
Female	1.00 [reference]	1.00 [reference]
Male	**1.48 [1.05, 2.09]**	1.42 [0.99, 2.04]
**Number of SARS-CoV-2 tests***	**1.16 [1.11, 1.21]**	**1.18 [1.13, 1.24]**
**Education**		
Elementary school or lower	1.00 [reference]	1.00 [reference]
Middle school	1.19 [0.77, 1.84]	1.17 [0.75, 1.85]
High school	0.76 [0.44, 1.32]	0.81 [0.46, 1.42]
Professional High school	**2.07 [1.04, 4.13]**	1.93 [0.92, 4.04]
University	**2.24 [1.28, 3.91]**	1.55 [0.84, 2.86]
Other or no school	**3.27 [1.73, 6.18]**	1.80 [0.83, 3.78]
**Highest parents' education**		
University or higher	1.00 [reference]	1.00 [reference]
High school	1.21 [0.85, 1.72]	1.21 [0.83, 1.76]
Middle school or lower	**3.61 [1.81, 7.22]**	**3.81 [1.84, 7.88]**
**Parents' Workplace**		
Home	1.00 [reference]	1.00 [reference]
At least partially outside	**1.58 [1.00, 2.49]**	**1.63 [1.00, 2.67]**

Abbreviations: CI = Confidence Interval; OR = Odds Ratio
Note: significant Odds Ratios are in bold
*N=18 missing. Reported Odds Ratio by unit increase

Supplementary Table S3: Multivariable analysis for the risk of at least one person among cohabitants tested positive for SARS-CoV-2 in the overall study population and in the subgroup of athletes enrolled in sports centers. Analysis comparing families of with a different number of weekly training sessions.

	**Overall Analysis (n=1,730)**		**Enrolled in Sport Centers (n=1,668)**	
	**N (% at least one positive test among cohabitants)**	**OR [95% CI]**	**N (% at least one positive test among cohabitants)**	**OR [95% CI]**
**Weekly training sessions**				
No	488 (18%)	1.00 [reference]	488 (18%)	1.00 [reference]
Up to two	850 (18%)	1.08 [0.78, 1.49]	804 (17%)	0.92 [0.66, 1.28]
More than two	392 (20%)	0.93 [0.63, 1.37]	376 (17%)	0.70 [0.46, 1.06]
**Gender**				
Female	772 (17%)	1.00 [reference]	741 (16%)	1.00 [reference]
Male	934 (19%)	1.21 [0.92, 1.59]	903 (18%)	1.19 [0.89, 1.58]
**Geographical area**				
North	1,116 (18%)	1.00 [reference]	1,082 (17%)	1.00 [reference]
Center	273 (14%)	**0.61 [0.41, 0.92]**	267 (14%)	**0.65 [0.43, 0.99]**
South and Islands	270 (19%)	1.14 [0.79, 1.63]	255 (17%)	1.02 [0.69, 1.50]
Other	29 (24%)	1.02 [0.38, 2.79]	27 (26%)	1.21 [0.44, 3.32]
**Number of SARS-CoV-2 tests***	-	**1.20 [1.15, 1.25]**	-	**1.21 [1.16, 1.27]**
**Education**				
Elementary school or lower	717 (14%)	1.00 [reference]	691 (13%)	1.00 [reference]
Middle school	424 (21%)	1.25 [0.90, 1.76]	410 (21%)	1.30 [0.92, 1.84]
High school	289 (12%)	**0.63 [0.40, 0.98]**	284 (12%)	0.65 [0.41, 1.03]
Professional High school	78 (29%)	**1.86 [1.05, 3.31]**	75 (28%)	**1.92 [1.06, 3.50]**
University	147 (24%)	**1.64 [1.01, 2.67]**	141 (21%)	1.30 [0.77, 2.19]
Other or no school	72 (40%)	**3.06 [1.72, 5.43]**	65 (34%)	**2.13 [1.13, 4.02]**
**Number of cohabitants***	-	**1.17 [1.02, 1.33]**	-	**1.16 [1.01, 1.33]**
**Highest parents' education**				
University or higher	1,102 (17%)	1.00 [reference]	1,067 (16%)	1.00 [reference]
High school	555 (20%)	1.14 [0.86, 1.52]	533 (18%)	1.13 [0.84, 1.52]
Middle school or lower	48 (38%)	**2.29 [1.18, 4.45]**	45 (36%)	**2.26 [1.13, 4.52]**
**Parents' Workplace**				
Home	339 (14%)	1.00 [reference]	328 (12%)	1.00 [reference]
At least partially outside	1,369 (19%)	**1.59 [1.11, 2.29]**	1318 (18%)	**1.67 [1.14, 2.44]**

Abbreviations: CI = Confidence Interval; OR = Odds Ratio
Note: significant Odds Ratios are in bold
* N=18 missing. Reported Odds Ratio by unit increase

Supplementary Table S4: Multivariable analysis for the risk of being tested positive for SARS-CoV-2 only for athletes who participated at least in one training session during the study period. Analysis comparing athletes with training sessions in opening and closing periods.

	N (% at least one positive test)	OR [95% CI] (N=1,236)
**Training by closure period^**		
At least one in opening period	936 (11%)	1.00 [reference]
At least one in closure period	300 (10%)	0.83 [0.50-1.36]
**Gender**		
Female	513 (9%)	1.00 [reference]
Male	708 (11%)	1.50 [0.92-2.43]
**Number of SARS-CoV-2 tests***		**1.22 [1.16-1.29]**
**Education**		
Elementary school or lower	522 (7%)	1.00 [reference]
Middle school	334 (12%)	1.31 [0.77-2.23]
High school	215 (7%)	0.69 [0.33-1.42]
Professional High school	61 (18%)	2.11 [0.92-4.80]
University	61 (24%)	**2.40 [1.06-5.42]**
Other or no school	40 (30%)	2.37 [0.96-5.88]
**Highest parents' education**		
University or higher	796 (9%)	1.00 [reference]
High school	394 (12%)	1.24 [0.79-1.95]
Middle school or lower	26 (38%)	**3.31 [1.27-8.66]**
**Location of training sessions**		
Mainly indoor	467 (9%)	1.00 [reference]
Mainly outdoor	602 (12%)	1.42 [0.85-2.37]
Both indoor and outdoor	165 (12%)	1.80 [0.91-3.53]
**Individual or team sports**		
Trainings in team	875 (9%)	1.00 [reference]
Individual training with other persons in the same space	262 (14%)	**1.68 [1.04-2.74]**
Individual training without other persons in the same space	99 (16%)	1.45 [0.73-2.87]
**Contact Sports**[∞]		
Yes	570 (10%)	1.00 [reference]
No	661 (11%)	1.44 [0.88-2.34]
**Triage**		
No	214 (26%)	1.00 [reference]
Yes	1022 (7%)	**0.33 [0.20-0.54]**
**No use of lockers and showers**		
Yes	245 (23%)	1.00 [reference]
No	991 (7%)	**0.53 [0.32-0.88]**
**Distancing**		
No	243 (19%)	1.00 [reference]
Yes	993 (9%)	**0.57 [0.35-0.93]**
**Continuous areation**		
No	756 (13%)	1.00 [reference]
Yes	480 (7%)	**0.59 [0.32-1.00]**

Abbreviations: CI = Confidence Interval; OR = Odds Ratio
Note: significant Odds Ratios are in bold
^Opening periods: September/October 2020 and April/May 2021; closure period: November 2020 to March 2021
*By unit increase
[∞]Contact sports: soccer, basketball, volleyball, martial arts, rugby, water polo, water basket, couple dance. No contact sports: swimming, gymn, dance, athletics, tennis, equitation, skating, climbing, skateboard, parkour, water aerobics, pilates, yoga, running, trekking, walking.

Supplementary Figure S3. Percentage of SARS-CoV-2 positive tested athletes according to type of sport.

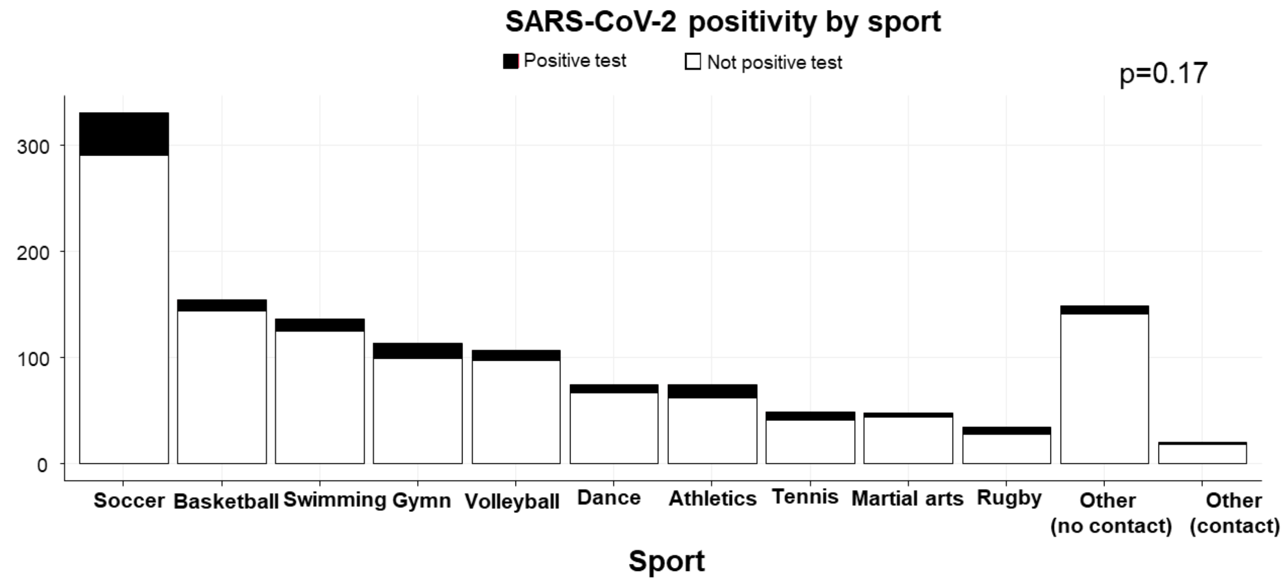

Supplementary Figure S4. Box plots of BMI percent change of athletes according to the number of participation of weekly training sessions, for all sample (N=1,475) and stratified by overweight and normal weight at the baseline.

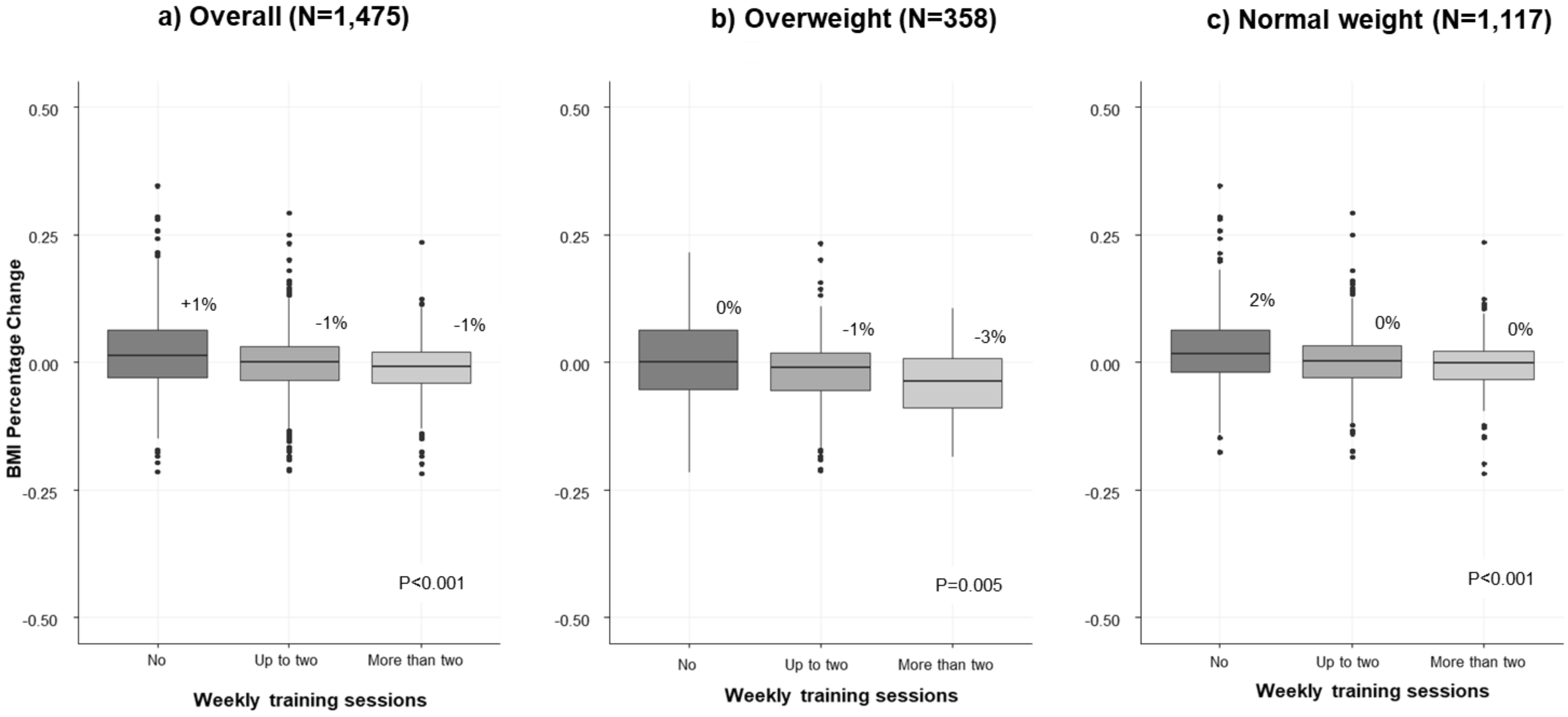

Supplementary Table S5. Multivariable analysis for the logarithm of the change of Body Mass Index (BMI) according to training sessions and other cofactors.

	N (%)	Beta [95% CI]
**Training by closure period^**		
At least one in closure period	805 (55%)	0.00 [reference]
At least one in opening period	255 (17%)	0.00 [-0.01, 0.01]
No	415 (28%)	**0.01 [0.00, 0.02]**
**Pre Covid BMI***	1475*	**0.90 [0.88, 0.92]**
**Gender**		
Female	683 (47%)	0.00 [reference]
Male	773 (53%)	**-0.01 [-0.01, 0.00]**
**Education**		
Elementary school or lower	597 (41%)	0.00 [reference]
Middle school	351 (24%)	-0.01 [-0.02, 0.00]
High school	266 (18%)	**0.01 [0.00, 0.02]**
Professional High school	63 (4%)	**0.01 [0.00, 0.03]**
University	130 (9%)	**0.03 [0.02, 0.04]**
Other or no school	67 (5%)	**0.03 [0.01, 0.05]**
**Geographical area**		
North	963 (66%)	0.00 [reference]
Center	248 (17%)	0.00 [-0.01, 0.01]
South and Islands	216 (15%)	**0.01 [0.00, 0.02]**
Other	30 (2%)	-0.01 [-0.03, 0.01]
**Adherence to WHO guidelines for physical activity**		
No	966 (67%)	0.00 [reference]
Yes	476 (33%)	**-0.01 [-0.01, 0.00]**
**Outdoor physical activity**		
No	466 (32%)	0.00 [reference]
Yes	1009 (68%)	**-0.01 [-0.01, 0.00]**
**Daily screen time**		
≤2 hours	345 (27%)	0.00 [reference]
2-4 hours	346 (27%)	**0.01 [0.00, 0.02]**
≥4 hours	573 (45%)	**0.02 [0.01, 0.02]**

Abbreviations: CI = Confidence Interval

Note: significant coefficients are in bold

^Opening periods: September/October 2020 and April/May 2021; closure period: November 2020 to March 2021

*By unit increase. Log transformation.

Supplementary Table S6. Multivariable analysis for the probability of physical inactivity, defined on the basis of the WHO guidelines, according to training sessions and other cofactors.

	N (% non-adherence to WHO guidelines)	OR [95% CI]
**Training by closure period^**		
At least one in closure period	928 (60%)	1.00 [reference]
At least one in opening period	294 (76%)	**1.51 [1.09, 2.10]**
No	478 (77%)	**2.15 [1.54, 3.02]**
**Gender**		
Female	767 (66%)	1.00 [reference]
Male	908 (69%)	0.86 [0.66, 1.12]
**Education**		
Elementary school or lower	151 (36%)	1.00 [reference]
Middle school	678 (76%)	**9.44 [6.10, 14.8]**
High school	421 (71%)	**7.87 [5.01, 12.5]**
Professional High school	296 (64%)	**4.99 [3.15, 7.99]**
University	75 (64%)	**6.06 [3.21, 11.6]**
Other or no school	75 (44%)	1.64 [0.89, 3.02]
**Contact Sports**		
No	935 (64%)	1.00 [reference]
Yes	694 (70%)	**1.39 [1.07, 1.81]**
**Matches or competitions**		
Yes	497 (52%)	1.00 [reference]
No	1203 (73%)	**1.91 [1.47, 2.48]**
**Outdoor space at home**		
Yes	956 (62%)	1.00 [reference]
No	744 (74%)	**1.61 [1.27, 2.03]**
**Outdoor physical activity**		
Yes	1159 (63%)	1.00 [reference]
No	541 (77%)	**1.46 [1.11, 1.92]**
**Daily screen time**		
≤2 hours	425 (60%)	1.00 [reference]
2-4 hours	387 (71%)	**1.53 [1.11, 2.12]**
≥4 hours	650 (68%)	**1.84 [1.35, 2.52]**
**Mediterranean Diet**		
Adherence	510 (64%)	1.00 [reference]
No adherence	875 (71%)	**1.29 [1.00, 1.67]**

Abbreviations: CI = Confidence Interval; OR = Odds Ratio
Note: significant Odds Ratios are in bold
^Opening periods: September/October 2020 and April/May 2021; closure period: November 2020 to March 2021

References

1. Bell A. "Designing and testing questionnaires for children." *Journal of Research in Nursing* 12.5 (2007): 461-46.

2. Craig CL, Marshall AL, Sjöström M, et al. International physical activity questionnaire: 12-country reliability and validity. *Med Sci Sports Exerc* 2003; 35(8): 1381-95.

3. Gnagnarella P, Dragà D, Misotti AM, et al. Validation of a short questionnaire to record adherence to the Mediterranean diet: An Italian experience. *Nutr Metab Cardiovasc Dis* 2018; 28(11): 1140-7.

4. https://www.who.int/tools/growth-reference-data-for-5to19-years/indicators/bmi-for-age. Accessed April 2022.

5. https://www.who.int/publications/i/item/9789240015128. Accessed April 2022.
